# Dr. De Laskie Miller

**Published:** 1903-08

**Authors:** 


Dr. De Laskie Miller, of Chicago, died at his home in that city July 9, 1903, aged 85 years. He graduated from Geneva Medical College in 1842, and began the practice of medicine in Lockport, N. Y., removing from there to Flint, Mich., and finally settling at Chicago, where he resided 51 years. He had been a teacher in Rush Medical College for the greater part of that time. He was one of the most prominent and highly respected physicians in Chicago.

